# A Digital Twin Framework for Structural Health Monitoring of Existing Large-Span Bridges

**DOI:** 10.3390/s26113293

**Published:** 2026-05-22

**Authors:** Minh Quang Tran, Hélder S. Sousa, José C. Matos, Son N. Dang, Huan X. Nguyen

**Affiliations:** 1 ISISE, ARISE, Department of Civil Engineering, University of Minho, 4800-058 Guimarães, Portugal; hssousa@civil.uminho.pt (H.S.S.); jmatos@civil.uminho.pt (J.C.M.); sondn@civil.uminho.pt (S.N.D.); 2 Faculty of Science and Technology, Middlesex University, London NW4 4BT, UK; h.nguyen@mdx.ac.uk

**Keywords:** digital twin, structural health monitoring, large-span bridges, sparse sensing, virtual sensors

## Abstract

Large-span bridges are critical components of transportation networks. Environmental variability, material degradation, and cumulative fatigue continuously affect their long-term performance. Digital Twin (DT) technology has emerged as a promising paradigm for integrating sensing, modeling, and data analytics. Most existing DT implementations in civil infrastructure rely on dense sensor networks, assume near-complete observability, and primarily serve as passive visualization or diagnostic tools, limiting their scalability and practical applicability. This paper proposes a DT framework specifically designed for the monitoring and management of existing large-span bridges under sparse sensing conditions. The framework adopts an information-centric perspective in which limited physical measurements are complemented by full-field state reconstruction through the integration of physics-based modeling, data-driven learning, and uncertainty-aware inference. A synchronized reference configuration, termed State 0, is introduced as the initial basis for tracking structural changes over time, while allowing conditional re-baselining through a Dynamic State 0 (DS0) when verified reassessment justifies it. On this basis, the proposed DT is formulated as an adaptive and decision-oriented cyber–physical system that supports optimization-based recommendations for sensing, inspection, and maintenance planning.

## 1. Introduction

Large-span bridges play a critical role in modern transportation networks [1]. However, their long-term performance is continuously affected by environmental variability, material degradation, fatigue accumulation, and extreme events [2]. Ensuring the structural safety and serviceability of these systems over the course of decades remains a fundamental challenge. Traditional inspection-based approaches are labor-intensive, subjective, expensive, and discontinuous. Structural health monitoring (SHM) plays a key role in ensuring the safety and long-term durability of long-span bridges. These systems collect and analyze real-time sensor data. This process enables early detection and warning of potential damage and supports the assessment of structural performance. SHM systems enhance operational reliability and reduce the risk of failure under increasingly harsh service conditions [3].

Previous studies have demonstrated the effectiveness of SHM systems [4,5,6]. Early research focused on vibration-based techniques to identify changes in modal properties associated with structural damage [7]. Yan et al. [8] presented a wavelet-based method that uses free vibration responses to accurately localize structural damage. Perera et al. [9] proposed a set of multivariate genetic algorithm frameworks incorporating Pareto optimization and aggregation functions for damage detection in structural systems. Ashebo et al. [10] integrated in situ measurements with finite element models to assess the effect of main girder deflection on the transverse distribution of vehicular loads in bridge structures. More recent investigations have integrated wireless sensor networks, machine learning algorithms, and data-driven approaches to improve damage detection accuracy [11,12,13]. Several authors have reported that continuous monitoring enables the identification of deterioration processes at an early stage [14,15,16]. Other studies have emphasized the importance of SHM in supporting decision-making for maintenance and rehabilitation [17,18,19]. SHM systems provide a reliable framework for enhancing structural safety and serviceability [20].

In recent years, Digital Twin (DT) technology has been increasingly adopted as a promising solution for integrating sensing, modeling, and data analytics into a unified cyber–physical framework [21,22,23]. A DT is commonly defined as a dynamic virtual representation of a physical asset that continuously synchronizes with real-world observations [24,25]. In principle, DTs enable real-time condition assessment, anomaly detection, and predictive maintenance [26,27,28]. In SHM applications, a DT can be initialized using limited sensor data and progressively refined through monitoring data and model updating. This enables structural condition assessment, potential damage prediction, and maintenance support. As a result, DT technology can improve operational safety while reducing unnecessary inspection and repair costs. Hoa et al. [29] and Long et al. [30] employed structural vibration parameters to update and develop numerical bridge models using optimization techniques. Changsu Shim et al. [31] and Son Dang et al. [32] applied Building Information Modeling (BIM) to establish DT frameworks. However, their approaches were limited to information management and did not incorporate structural performance data required for effective structural health monitoring. Several other studies [33,34,35] have also investigated the use of digital twins in SHM, but these approaches remain incomplete. Although the proposed models demonstrate high efficiency and accuracy, they typically represent only a single state of the structure. The virtual model may precisely replicate the physical object, but it often lacks real-time interaction with the actual structure. A true digital twin requires continuous data exchange and dynamic interaction between the physical and virtual models. Advances in the Internet of Things, cloud computing, and smart sensors have made integrated bridge DT systems increasingly feasible.

While BIM, SHM, and Digital Twins are often mentioned together in the context of infrastructure digitalization, they differ fundamentally in their objectives, temporal characteristics, and decision-making capabilities. BIM primarily serves as a static or discretely updated information repository, whereas SHM focuses on real-time condition monitoring based on sparse sensor measurements. In contrast, a Digital Twin aims to maintain a continuously synchronized, uncertainty-aware, and predictive representation of the physical system. Many reported DT implementations in civil engineering remain closely tied to relatively rich instrumentation, measured-location-based interpretation, or high-fidelity numerical models that implicitly benefit from a high level of observability [6]. This assumption severely limits the practical deployment of DTs for large-span bridges. Dense instrumentation is often economically infeasible, technically challenging, and difficult to maintain over long service periods. Furthermore, sensor failures, data gaps, and environmental disturbances further compromise the reliability of purely measurement-driven approaches. As a result, many current DT systems remain either static digital models or loosely coupled SHM platforms, rather than truly synchronized and adaptive cyber–physical systems.

Another recurring limitation in several bridge-oriented DT frameworks is that they primarily emphasize synchronization, visualization, monitoring, or diagnosis. Explicit prescriptive functions, such as uncertainty-driven sensor adaptation, inspection prioritization, and maintenance optimization, are less frequently formalized. Most digital twins aim to replicate the current state of the physical structure but lack mechanisms for systematic baseline definition, longitudinal comparison, uncertainty-aware inference, and closed-loop decision-making. Consequently, these systems often provide strong descriptive or diagnostic support, but their prescriptive capability depends on whether decision rules, optimization objectives, and uncertainty-aware action mechanisms are explicitly implemented.

Existing bridge DT studies have advanced several important aspects of infrastructure digitalization, including BIM/GIS-based asset integration, IoT-enabled SHM platforms, model updating, real-time monitoring, and visualization-based decision support [36,37]. However, the research gap at the intersection of these functions remains insufficiently addressed. Many frameworks still assume relatively rich sensing or restrict interpretation to measured locations [38]. Others provide model synchronization or lifecycle visualization but do not explicitly manage a reference baseline for longitudinal comparison [39]. Similarly, virtual sensing and full-field reconstruction are often not linked to uncertainty representation, while optimization and decision-support functions are frequently described conceptually rather than formulated as uncertainty-aware decision problems [40,41]. Consequently, there is a need for a DT framework that treats sparse sensing as a design condition, reconstructs unmeasured responses with uncertainty-awareness, treats State 0/DS0 as a reference for long-term comparison, and uses reconstructed-state uncertainty to support sensor placement, inspection prioritization, and maintenance planning.

To address these challenges, this paper proposes a new Digital Twin framework that explicitly departs from dense-sensing paradigms and adopts an information-centric perspective. The proposed DT is designed to operate under sparse physical sensing conditions, where only a limited number of strategically placed sensors are deployed. Missing information is compensated through virtual sensor reconstruction, which integrates physics-based models, data-driven learning, and probabilistic inference to estimate unmeasured structural responses.

A key innovation of the proposed framework is the introduction of a synchronized reference configuration, termed State 0. In this study, State 0 is not a permanently fixed pristine baseline. It is a validated reference condition of the bridge at a given stage of operation. Subsequent states are evaluated relative to this reference, enabling consistent longitudinal assessment. For long-term monitoring, State 0 may be conditionally updated into a Dynamic State 0 (DS0) after qualified reassessment, such as a validated load test, major intervention, or significant sensing reconfiguration. This mechanism keeps the DT adaptive while preserving a stable temporal anchor for degradation tracking and anomaly detection.

Furthermore, the proposed DT is conceived as an active and adaptive system. Based on reconstructed full-field states and uncertainty distributions, the DT continuously evaluates the effectiveness of the current sensing configuration. It formulates and solves information-driven optimization problems to guide sensor placement, inspection scheduling, and maintenance planning. In this way, the DT no longer acts as a passive digital mirror, but as a proactive decision-support agent.

A human-centered DT interface is developed. It visualizes reconstructed states, uncertainty representations, baseline deviations, and optimized recommendations in an interpretable form. This interface enables a closed-loop human–DT collaboration in which computational intelligence and engineering judgment complement each other. The main contributions of this paper are summarized as follows:

(i) A sparse-sensing-aware Digital Twin framework that reduces dependence on dense sensor networks through virtual sensor reconstruction and information-centric inference.
(ii) A synchronized reference-state concept, including State 0 and conditionally updateable Dynamic State 0 (DS0), for consistent assessment of structural changes over time.
(iii) A closed-loop DT architecture that integrates reconstruction, optimization, and decision support into a unified system.
(iv) A human-centered interface concept aimed at improving the interpretability and practical usability of Digital Twin-assisted bridge management.

The remainder of this paper is organized as follows. Section 2 reviews the fundamentals of Digital Twin technology and related concepts. Section 3 presents the proposed framework in detail. Section 4 discusses enabling technologies and methodological implementation. Section 5 presents a component-level proof-of-concept case study. Section 6 outlines challenges and future research directions. Finally, Section 7 concludes the paper.

## 2. Understanding Digital Twin Technology

In the context of civil infrastructure, DTs promise to provide a holistic, continuous representation of structural systems throughout their lifecycles. This section clarifies the fundamental principles of Digital Twin technology, distinguishes DTs from related concepts, and introduces a maturity-oriented perspective. These discussions establish the conceptual foundation for the proposed framework in Section 3.

### 2.1. Definition and Core Principles of Digital Twins

A DT can be broadly defined as a continuously evolving digital representation of a physical system that remains synchronized with its real-world counterpart through data-driven and model-based mechanisms. Unlike conventional simulations, which are typically executed offline and independently of real-world operations, a DT operates as a persistent computational entity that updates its internal state in response to incoming information.

**Figure 1:**
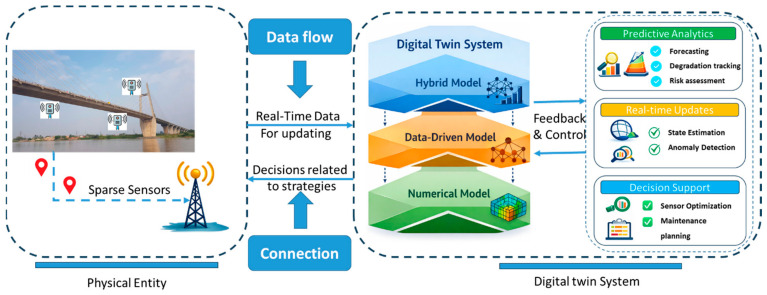
Digital twin for Bridge monitoring.

Three core components characterize a DT system: the physical entity, the digital representation, and the cyber–physical connection. The physical entity corresponds to the real-world system, such as a bridge, its components, and its operational environment. The digital representation comprises a hierarchy of models, including geometric descriptions, physics-based behavioral models, reduced-order representations, and data-driven surrogates. The cyber–physical connection enables continuous information exchange between these two domains (Figure 1).

A defining characteristic of a digital twin is its statefulness. Instead of producing isolated outputs for given inputs, a DT maintains an internal state that evolves over time. This state may include directly observable quantities, such as accelerations or displacements, as well as latent variables, such as stiffness degradation, residual stresses, or damage indicators. Maintaining such an internal state allows the DT to support temporal reasoning, anomaly detection, and trend analysis.

Another essential principle of DT systems is awareness of uncertainty. Real-world measurements are inevitably affected by noise, missing data, and sensor malfunctions, whereas numerical models are subject to epistemic errors arising from simplifications and unknown parameters. A credible DT must therefore incorporate mechanisms for representing, propagating, and updating uncertainty. Without explicit uncertainty modeling, DT predictions can easily become misleading, especially in long-term monitoring scenarios [42,43,44].

Beyond state estimation, a DT is expected to support predictive and prescriptive functionalities. Predictive capabilities enable the forecasting of future system behavior, while prescriptive capabilities allow the DT to recommend actions that improve system performance or safety.

This observation motivates the need for DT frameworks that move beyond passive representation and explicitly integrate inference, optimization, and feedback mechanisms. The proposed framework in this paper adopts this perspective by treating the DT as an adaptive information-processing system rather than a static digital replica.

### 2.2. Digital Twin Versus Related Concepts: BIM, SHM, and Smart Infrastructure

DT technology is often discussed in conjunction with related concepts, including Building Information Modeling (BIM), Structural Health Monitoring (SHM), and smart infrastructure systems. While these paradigms share certain common elements, such as data integration and digital representation, they differ fundamentally in their objectives, operational logic, and level of intelligence.

BIM primarily focuses on the management of geometric, semantic, and lifecycle information of built assets. It provides a centralized repository for design specifications, construction records, and maintenance documentation. However, BIM models are typically static or manually updated and do not inherently support continuous synchronization with the physical structure. As a result, BIM serves as an information management platform rather than a dynamic representation of structural behavior.

Traditional SHM systems, on the other hand, emphasize the acquisition and processing of sensor data to detect anomalies, identify modal properties, or localize damage. SHM has significantly advanced the understanding of structural behavior under real operating conditions. Nevertheless, most SHM implementations treat sensor data as isolated observations rather than as components of a continuously evolving digital state. The collected data are analyzed but rarely integrated into a persistent, adaptive digital model. Consequently, SHM systems are largely diagnostic, focusing on what has happened rather than what is likely to happen or what should be done.

Smart infrastructure systems integrate sensing, communication, and automation technologies to enhance operational efficiency. However, they often rely on rule-based or heuristic control strategies and typically lack a coherent and evolving digital representation of the underlying physical system. Their intelligence is frequently localized and reactive rather than system-level and predictive. Table 1 summarizes the main differences among BIM, SHM, smart infrastructure systems, and Digital Twin (DT) approaches.

Despite these conceptual advantages, many DT implementations in civil engineering remain closely aligned with SHM platforms or enhanced BIM models. They often emphasize visualization, data dashboards, and real-time updates, but lack systematic mechanisms for uncertainty-aware inference, identifying information gaps, and closed-loop optimization.

By explicitly assuming sparse sensing and embedding virtual sensor reconstruction into the core of the DT architecture, the proposed system treats incomplete observability as a design condition rather than a limitation. This shift enables the DT to function as an inference-driven and decision-oriented system rather than as a passive data mirror. Moreover, by integrating optimization and decision-support mechanisms, the proposed DT goes beyond conventional SHM and smart infrastructure systems. It not only monitors the structure but also reasons about how monitoring itself should be performed. This reflexive capability (monitoring the monitoring process) is a defining feature of advanced digital twin systems and a key motivation for the proposed framework.

### 2.3. Maturity Levels and Functional Capabilities of Digital Twins

Digital Twin systems can be classified by maturity, reflecting their degree of synchronization, autonomy, and intelligence. This maturity-oriented perspective clarifies both what a DT represents and what it can do.

At the lowest level, a digital model provides a static or quasi-static representation of the physical system. Such models may be highly detailed in terms of geometry and material properties, but they are not continuously synchronized with real-world observations. Once constructed, they remain largely unchanged unless manually recalibrated. Many conventional finite element models and design-stage simulations fall into this category.

At the second level, a synchronized digital twin incorporates periodic or real-time data assimilation. Sensor measurements are used to update model parameters and states, allowing the digital representation to reflect the current condition of the physical system. Most DT implementations in civil engineering belong to this category. They emphasize real-time visualization, model updating, and anomaly detection. However, these systems typically focus on representing the present state rather than reasoning about the future or influencing the monitoring process itself.

At the third level, a predictive digital twin extends synchronization by incorporating forecasting capabilities. Using historical data, degradation models, and machine learning, such DTs can predict future system behavior, estimate remaining useful life, and simulate hypothetical scenarios. These systems are no longer purely descriptive, but they still tend to operate in an open-loop manner. Predictions are generated, but they rarely influence how data should be collected or how the monitoring strategy should evolve.

At the highest maturity level, a digital twin becomes prescriptive and adaptive. In addition to synchronization and prediction, it actively reasons about uncertainty, information gaps, and decision outcomes. It continuously evaluates the effectiveness of its own sensing and modeling processes, and adapts them through optimization and feedback. In this sense, the DT no longer only answers “what is happening?” or “what will happen?”, but also “what should be done next?”. To further clarify the functional progression of Digital Twins, Table 2 summarizes the main maturity levels for description, capabilities, applications, advantages, and limitations. This classification shows that DT maturity is not solely a matter of model fidelity or synchronization frequency, but also of predictive capability, feedback integration, and decision relevance.

From this perspective, higher DT maturity is associated with the ability to operate under incomplete observability, support predictive reasoning, and contribute to closed-loop decision-making. These considerations motivate the framework introduced in Section 3.

## 3. Proposed Digital Twin Framework for Existing Large Bridge

### 3.1. Proposed Framework

**Figure 2:**
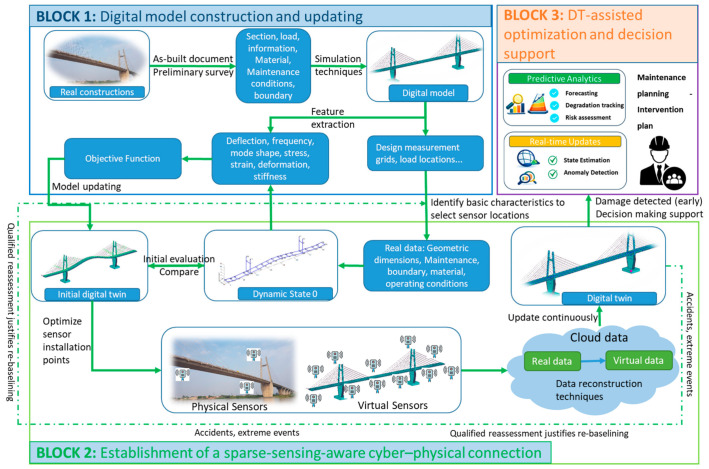
Proposed framework for Structural Health Monitoring of existing Large-span Bridge.

Building upon the principles discussed in Section 2, this section introduces a Digital Twin (DT) framework specifically designed for the long-term monitoring and management of existing large-span bridges. As illustrated in Figure 2, the framework is organized into three functional blocks that operate in a progressive and iterative manner: (i) digital model construction and updating, (ii) establishment of a sparse-sensing-aware cyber–physical connection, and (iii) DT-assisted optimization and decision support. These blocks are not isolated modules. They form a closed-loop system in which information circulates between the physical structure, the digital model, and human operators.

The first block focuses on constructing the initial digital representation of the bridge and establishing its synchronization with the physical system. This process integrates geometric information, material properties, boundary conditions, and physics-based behavioral models, and then data-driven updating refines them using available measurements. A major goal of this block is to develop a synchronized reference configuration, called State 0, that will serve as the starting point for future comparisons and long-term evaluations. For long-term operation, this reference may be conditionally updated into a Dynamic State 0 (DS0) when a qualified reassessment justifies re-baselining.

The second block establishes a stable cyber–physical connection under sparse sensing conditions. Instead of relying on dense sensor networks, only a limited number of strategically placed physical sensors are deployed. Their measurements are transmitted to a cloud-based platform, where they are fused with historical and simulation-based information. Based on this hybrid dataset, the DT reconstructs unmeasured responses through virtual sensor generation. This mechanism enables full-field state estimation while preserving economic and practical feasibility.

The third block transforms the DT from a synchronized representation into a decision-support system. Using reconstructed states and uncertainty estimates, it identifies information gaps, evaluates sensor effectiveness, and supports optimization of sensing, inspection, and maintenance actions. Sensor placement can be guided by information gain, uncertainty reduction, and damage sensitivity. Inspection and maintenance priorities can be derived from state deviations, uncertainty levels, predicted risk, and practical constraints. Thus, the DT acts not only as a synchronized digital representation, but also as an adaptive system that informs what should be measured, inspected, or maintained next.

To facilitate effective human-DT collaboration, a dedicated interface layer is integrated into the framework. This interface visualizes reconstructed full-field states, uncertainty-aware representations, baseline deviations, and optimized recommendations in a transparent and interpretable manner. Through this interaction layer, domain experts remain actively involved in the decision-making loop. By addressing sparse sensing, baseline-aware tracking, and closed-loop optimization, the framework provides a practical foundation for long-term management of large-span bridges.

### 3.2. Block 1: Digital World—Development and Updating of the Digital Model

**Figure 3:**
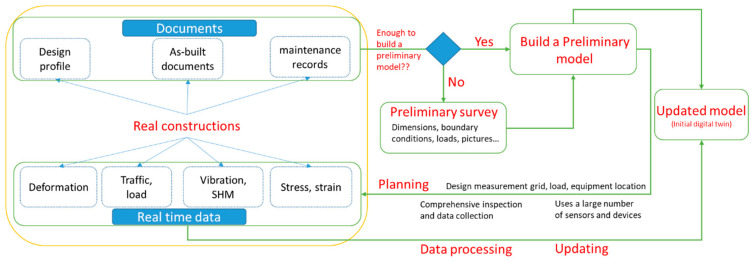
First block of DT framework.

The first block of the proposed framework focuses on the construction and continuous refinement of the digital representation of the bridge (Figure 3). This block establishes the computational foundation for all subsequent cyber–physical interactions, reconstructions, and optimizations. The objective is not to develop a perfect initial model. Instead, it is to build a structurally consistent model that can be progressively improved.

The process begins with the creation of an initial physics-based model. The essential characteristics of the bridge can be captured, including its geometry, material properties, boundary conditions, and loading scenarios. Depending on the required level of fidelity, the model may take the form of a finite element model, a reduced-order model, or a modal representation. This initial model provides a prior description of the structural behavior, which is subsequently refined through data assimilation.

As measurements from the physical system become available, the digital model is continuously updated to reduce the mismatch between simulated and observed structural responses. The mismatch is quantified through residuals defined in terms of measured quantities or derived response features, such as time-domain responses, modal frequencies, mode shapes, or strain distributions, depending on data availability and the monitoring objective. Model updating is then performed by minimizing these residuals under physically meaningful constraints, ensuring improved agreement with observations without compromising structural interpretability. This updating process may involve parameter identification, state estimation, or hybrid approaches that combine physics-based constraints with data-driven learning. The goal is not merely to fit the data, but to preserve physical interpretability while improving predictive consistency. A key outcome of this updating process is the emergence of a synchronized digital representation. The model can reflect the actual mechanical behavior of the structure at the time of observation. At this stage, the digital model no longer represents an idealized design-state structure but rather a data-informed digital entity that embodies the current condition of the bridge.

Concurrently, the physical structure begins to participate actively in the cyber–physical loop. By deploying sensors and data-acquisition mechanisms, the structure becomes capable of “perceiving” its own state and communicating this information to the digital domain. This mutual awareness between the digital and physical worlds marks the onset of a true Digital Twin relationship.

At the end of this block, the DT reaches an initial synchronized configuration composed of two coupled entities: an updated digital model in the virtual domain and the physical bridge connected through sensing and data transmission. The simulated responses are brought into consistency with measured structural behavior. This establishes a validated cyber–physical reference configuration, denoted as State 0. Later DT updates are interpreted relative to this reference state. This provides the temporal basis for long-term assessment of structural evolution. Accordingly, deviations observed at later times are evaluated not in absolute terms but as changes relative to the synchronized baseline. For long-term operation, this reference may be conditionally updated into a Dynamic State 0 (DS0) when a qualified reassessment justifies re-baselining.

Formally, let x(t) denote the reconstructed full-field state of the structure at time t, and x0 denote the reference state corresponding to State 0. Structural evolution is quantified by the deviation Δx(t) = x(t) − x_0_, thereby enabling consistent tracking of gradual degradation, abnormal changes, and long-term performance trends. To preserve interpretability, x_0_ is treated as a stable reference during routine monitoring rather than a continuously drifting baseline. Nevertheless, State 0 is not regarded as permanently fixed. Following major structural interventions, validated global reassessment, or substantial sensing and operational reconfiguration, the reference state may be conditionally updated into a DS0. Subsequent state deviations are then evaluated relative to the updated reference, while the transition between reference states is explicitly documented to maintain lifecycle consistency.

The transition from State 0 to DS0 should be governed by explicit re-baselining rules. State 0 is kept fixed during routine monitoring and is not automatically updated under normal environmental or operational variability. A new DS0 may be established only after a qualified reassessment event, such as major repair, structural strengthening, validated load testing, significant sensor reconfiguration, or engineering inspection confirming that the structure has entered a new reference condition.

The candidate DS0 should be accepted only when the updated model and reassessment measurements satisfy predefined validation criteria. These criteria may include model–measurement agreement, modal consistency, serviceability compliance, and short-term stability of reconstructed state deviations. Representative indicators include modal frequency error, MAC, serviceability demand ratio, and deviation variability over a stabilization window. The corresponding thresholds should be selected according to bridge type, sensor accuracy, design codes, owner requirements, baseline statistics, and reliability-informed engineering judgment.

Once accepted, the transition must be documented by recording the previous State 0, the new DS0, the reassessment event, the validation metrics, and the transition time. Future deviations are then evaluated relative to the active DS0, while previous reference states remain archived for lifecycle traceability. This event-governed procedure prevents the reference baseline from drifting with gradual deterioration while still allowing justified re-baselining after verified structural or monitoring-system changes.

In addition to serving as a relative reference for tracking structural evolution, State 0 can also be linked to serviceability limit state (SLS) criteria for practical decision-making. In this interpretation, the DT evaluates not only the deviation from the reference state, but also the margin between the reconstructed response and allowable serviceability thresholds. Typical SLS indicators may include vertical deflection, acceleration or vibration comfort indices, strain or stress ranges, crack-related indicators when available, and modal-frequency shifts. For example, for a reconstructed deflection response ${\hat{\delta }}_{j}(t)$ at location *j*, the serviceability margin can be defined as:

$$g_{j}^{SLS}(t)=\delta_{j,\lim}-\left|{\hat{\delta }}_{j}(t)\right|$$

where $\delta_{j,\lim}$ is the allowable deflection threshold specified by design codes, owner requirements, or reliability-informed serviceability assessment. A corresponding demand ratio can be written as

$$\rho_{j}^{SLS}(t)=\frac{\left|{\hat{\delta }}_{j}(t)\right|}{\delta_{j,lim}}$$

A component is considered serviceability-critical when $\rho_{j}^{SLS}(t)\geq 1$ or when $\rho_{j}^{SLS}(t)$ shows a persistent increasing trend relative to State 0/DS0. Similarly, vibration- or strain-based serviceability indicators can be evaluated by replacing ${\hat{\delta }}_{j}(t)$ and $\delta_{j,lim}$ with the corresponding reconstructed response feature and allowable threshold. In this way, State 0 provides the baseline for detecting abnormal evolution, while SLS thresholds provide practical criteria for maintenance prioritization and intervention planning.

Block 1 establishes a synchronized, continuously updatable digital representation of the bridge and defines a reference baseline that serves as the foundation for subsequent inference, reconstruction, and optimization. This block transforms the digital model from a static artifact into a living entity whose state evolves in parallel with the physical structure. The enabling technologies and methodological components underlying model updating, cyber–physical synchronization, and reference-state establishment are discussed in Section 4.

### 3.3. Block 2: Real World—Establishing the Cyber–Physical Connection

**Figure 4:**
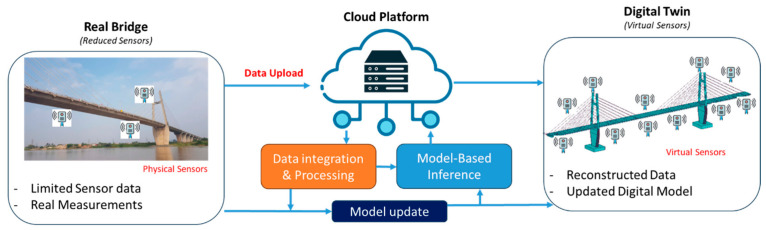
Real World—Establishing the Cyber–Physical Connection.

The second block of the proposed framework establishes a stable, scalable, and information-efficient cyber–physical connection between the digital twin and the real bridge (Figure 4). Conventional monitoring systems aim to maximize spatial coverage. They do this by deploying dense sensor networks. In contrast, this block explicitly adopts a sparse-sensing paradigm. Only a limited number of strategically placed sensors are installed on the physical structure. The missing information is compensated for through digital reconstruction and inference.

In practice, the selection of physical sensor locations is guided by structural sensitivity, modal participation, accessibility, and long-term maintainability. The system focuses on capturing a minimal yet informative subset of observations. These measurements are continuously transmitted to a cloud-based platform. The data are stored, processed, and fused with historical records and simulation-generated data.

Once uploaded to the cloud, the real-world measurements serve as inputs to a suite of physics-informed and data-driven reconstruction models. These models infer the responses at unmeasured locations and for uninstrumented components, effectively generating a set of virtual sensor signals. The DT maintains an expanded sensing layer that combines physical measurements with reconstructed quantities.

Based on a hybrid information stream, the DT reconstructs structural responses at unmeasured locations and for uninstrumented components, thereby generating virtual sensing layers that complement the physical measurements. Let $y_{S}(t)\in R^{m}$ denote the vector of measurements collected from the sparse physical sensor set S at time t, m ≪ n and n is the number of response quantities required to describe the structural state. The reconstructed structural state is obtained through a reconstruction operator R(⋅):

$$\left(\hat{x}(t),\sum_{x}\left(t\right))=R(y_{S}(t),M_{0},H,z(t);\theta_{R}\right)$$

where $\hat{x}(t)\in R^{n}$ is the reconstructed structural state, $\sum_{x}\left(t\right)$ denotes the associated reconstruction uncertainty, $M_{0}$ is the synchronized reference model associated with State 0, H represents historical monitoring data, *z*(*t*) denotes relevant environmental or operational variables and $\theta_{R}$ represents the parameters of the selected reconstruction model.

The operator R(⋅) can be instantiated using different methods depending on data availability, structural complexity, and computational requirements. Possible implementations include modal expansion, Kalman-type state estimation, Gaussian Process Regression, reduced-order model updating, physics-informed neural networks, and deep-learning-based reconstruction models. In the proof-of-concept case study presented in Section 5, R(⋅) is implemented using a hybrid 1DCNN–LSTM model, where the 1DCNN component extracts local temporal and inter-channel features from sparse sensor signals, and the LSTM component captures temporal dependencies in the dynamic response. This choice is supported by the authors’ previous reconstruction study, in which missing sensor channels in the composite plate dataset were reconstructed from the remaining measured channels and validated using MAE, modal frequency error, and MAC.

The uncertainty term $\sum_{x}\left(t\right)$ is introduced to avoid treating reconstructed virtual sensor outputs as deterministic substitutes for direct physical measurements. In the proposed framework, $\sum_{x}\left(t\right)$ summarizes the combined effects of measurement noise, model and parameter uncertainty, environmental or operational variability, and reconstruction error. It may be interpreted as

$$\sum_{x}\left(t\right)\approx \sum_{meas}\left(t\right)+\sum_{\mathrm{mod}el}\left(t\right)+\sum_{env}\left(t\right)+\sum_{rec}\left(t\right)$$

Depending on the selected implementation of R(⋅), these terms may be estimated through sensor calibration data, model-updating residuals, baseline environmental statistics, prediction covariance, Bayesian posterior variance, ensemble dispersion, Monte Carlo dropout, or validation residuals. In the present proof-of-concept, full probabilistic uncertainty propagation is not implemented. Instead, reconstruction reliability is assessed empirically by comparing reconstructed responses with hidden measured channels, while MAC degradation under increasing sensor loss is used as an indicator of information insufficiency.

This reconstruction process enables the digital twin to maintain a high-resolution representation of the structural state, even when the physical sensing density is low. The reconstructed quantities are not treated as deterministic values, but are associated with uncertainty measures that reflect data noise, model imperfections, and information loss due to sparse sensing. The reconstructed full-field state, together with the physical sensor measurements, is then used to update the digital model in Block 1. This continuous update ensures that the digital twin remains synchronized with the physical structure, not only at the measured locations but across the entire structural domain.

A key feature of this block is that the cyber–physical connection is not solely based on direct measurements. Instead, it relies on a hybrid information stream that integrates physical observations and virtual sensor reconstructions. This hybrid paradigm significantly reduces the dependence on dense sensor networks while preserving observability at the system level.

Moreover, this block is designed to be robust to data gaps, sensor failures, and intermittent connectivity. When physical data are temporarily unavailable, the digital twin can propagate the state forward using predictive models and historical patterns, thereby maintaining operational continuity.

Through this mechanism, the physical and digital domains become tightly coupled. The physical structure informs the digital twin through sparse but informative measurements, while the digital twin compensates for missing information through inference and reconstruction. The enabling methods underlying this reconstruction process are discussed in Section 4. This bidirectional interaction forms a stable cyber–physical loop that serves as the foundation for optimization and decision support in the next block.

Block 2 transforms sparse physical measurements into a rich and uncertainty-aware digital representation. By leveraging cloud-based computation and virtual sensor reconstruction, it ensures that the digital twin remains synchronized, scalable, and economically feasible for long-term deployment on large-span bridges.

### 3.4. Block 3: DT-Assisted Optimization and Decision Support Under Sparse Sensing

**Figure 5:**
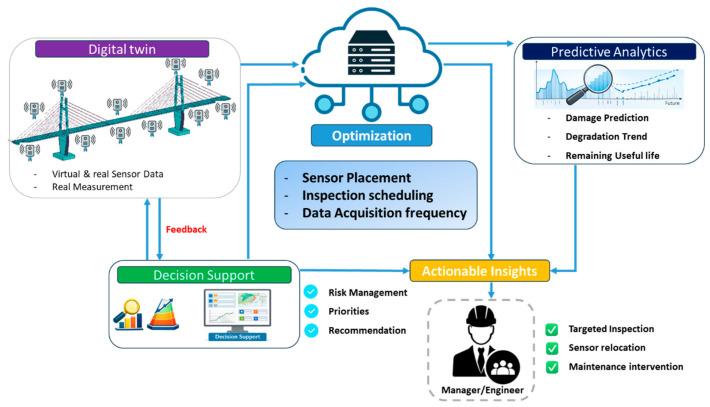
DT-Assisted Optimization and Decision Support under Sparse Sensing.

The third block of the proposed framework represents the transition from a synchronized digital representation to an active decision-support system (Figure 5). Blocks 1 and 2 focus on model construction, updating, and cyber–physical synchronization. Block 3 uses reconstructed states and uncertainty information to guide resource allocation, monitoring, and maintenance planning. A central assumption of the proposed framework is that sensing resources are limited. Instead of increasing the number of physical sensors, the Digital Twin seeks to maximize the informational value of the existing sensing infrastructure. This is achieved by analyzing the reconstructed state, its uncertainty, and its sensitivity to possible damage scenarios.

Let $\hat{x}(t)$ denote the reconstructed full-field state at time t and $\sum_{x}\left(t\right)$ denote its associated uncertainty representation. These quantities form the primary inputs to the optimization layer. Based on these quantities, the DT evaluates how well different regions of the structure are observed, which components are most critical to system-level behavior, and where information loss is most significant. To make this optimization concept explicit, a representative sensor placement problem can be formulated as an uncertainty-minimization problem. Let C denote the set of candidate sensor locations and let $s_{i}\in \left\{0,1\right\}$ be a binary decision variable, where *s_i_* = 1 indicates that a physical sensor is installed at candidate location I and *s_i_* = 0 otherwise. For a selected sensor configuration s, the reconstruction operator introduced in Block 2 provides a reconstructed state $\hat{x}(t)$ and an associated uncertainty representation $\sum_{x}\left(t;s\right)$. The sensor placement problem can then be expressed as

$$\underset{s}{\min}\;J(s)=tr(W\sum_{x}\left(s\right))+\lambda_{c}\sum_{i\in c}c_{i}s_{i}$$

subject to

$$\sum_{i\in c}s_{i}\leq N_{\max},\;\sum_{i\in c}c_{i}s_{i}\leq B,\;s_{i}\leq a_{i},\;s_{i}\in \left\{0,1\right\}$$

Here $\sum_{x}\left(s\right)$ denotes the time-averaged reconstruction uncertainty associated with the selected sensor configuration, W is a weighting matrix used to emphasize structurally critical or damage-sensitive response components; c_i_ is the installation and maintenance cost of a sensor at location I, Nmax is the maximum allowable number of sensors, B is the available budget and $a_{i}\in \left\{0,1\right\}$ indicates whether the candidate location is physically accessible and feasible for installation. The first term minimizes the uncertainty of the reconstructed structural state, while the second term penalizes sensing cost.

Alternatively, when an information matrix F(s) can be constructed from the selected sensor configuration, prior model information, and measurement-noise statistics, an information-gain formulation such as D-optimality may be adopted:

$$\underset{s}{\max}\log\det(F(s)+\varepsilon I)-\lambda_{c}\sum_{i\in C}c_{i}s_{i}$$

subject to the same cost, number, and feasibility constraints. The resulting constrained binary optimization problem may be solved using a sequential greedy sensor placement strategy, in which sensors are added according to the largest marginal reduction in reconstruction uncertainty or the largest marginal increase in information gain. For more complex bridge configurations, population-based methods such as Genetic Algorithms or Particle Swarm Optimization may also be used.

In addition to sensing optimization, the reconstructed states and their uncertainty can be translated into quantitative decision-support metrics. Let ${\hat{x}}_{j}(t)$ denote the reconstructed response feature of component or location j and $x_{0,j}$ be the corresponding value in the active State 0/DS0 reference. A normalized deviation indicator is defined as

$$d_{j}(t)=\frac{\left|{\hat{x}}_{j}(t)-x_{0,j}\right|}{\sigma_{0,j}+\varepsilon }$$

where $\sigma_{0,j}$ denotes the baseline variability and ε is a small regularization term. The associated uncertainty is obtained from the diagonal term of the reconstruction or prediction covariance matrix,

$$u_{j}(t)=\sqrt{\sum_{x,jj}\left(t\right)}$$

A representative priority score can then be expressed as

$$R_{j}(t)=\omega_{d}d_{j}(t)+\omega_{u}\frac{u_{j}(t)}{u_{j,ref}+\varepsilon }+\omega_{c}c_{j}$$

where $c_{j}$ is a component criticality factor, $u_{j,ref}$ is a reference uncertainty level, and $\omega_{d},\omega_{u},\omega_{c}$ are weighting coefficients. Risk trajectories are obtained by forecasting $R_{j}(t)$ over a prediction horizon T_P_. Components can then be ranked according to the maximum predicted risk, cumulative risk, risk growth rate, or probability of exceeding a predefined threshold:

$$\mathrm{Pr}(\left|{\hat{x}}_{j}(t+\tau )-x_{o,j}\right|>\delta_{j})>\alpha ,\;\tau \in [1,T_{P}]$$

where $\delta_{j}$ is an allowable deviation threshold and α is a prescribed confidence level. This formulation converts uncertainty-aware reconstructed states into transparent inspection and maintenance priorities.

Serviceability margins such as $g_{j}^{SLS}(t)$ and demand ratios such as $\rho_{j}^{SLS}(t)$ can be incorporated into the priority score, so that components are ranked not only by deviation from State 0/DS0, but also by their proximity to practical serviceability limits.

Another defining feature of this block is its adaptive nature. Decisions made based on DT recommendations (such as relocating a sensor, increasing sampling frequency, or conducting a targeted inspection) alter the information landscape of the system. These actions generate new data, which are fed back into Blocks 1 and 2, triggering further model updating and reconstruction. Over time, this feedback loop enables the DT to learn from its own decisions and progressively improve its performance.

This block, therefore, establishes a closed-loop control structure. The DT does not merely observe the system but actively shapes the monitoring process. The objective is not to maximize data volume, but to maximize actionable knowledge under resource constraints. Block 3 transforms the digital twin from a synchronized representation into an intelligent agent capable of reasoning about uncertainty, predicting future behavior, and optimizing sensing and maintenance strategies. This shift is fundamental to the proposed framework, as it enables scalable, cost-effective, and long-term deployment in real-world bridge management scenarios.

### 3.5. Digital Twin Interface: Human–DT Interaction and Decision-Support Layer

**Figure 6:**
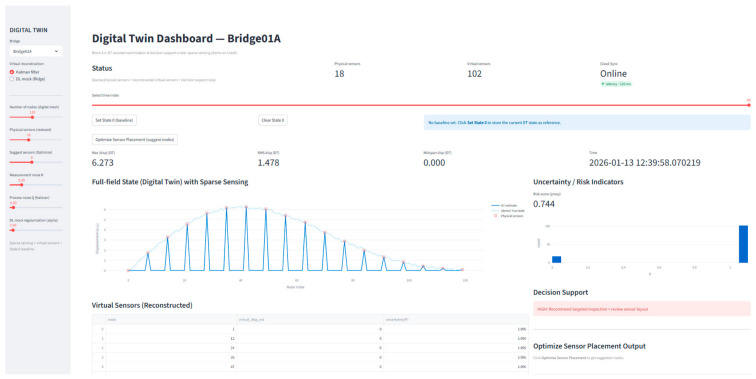
Proposed DT interface.

Within the proposed framework, the DT interface serves as the operational front-end that connects the underlying cyber–physical system with human decision-makers. The interface transforms the computational processes into interpretable, actionable, and trustworthy information (Figure 6). It is therefore not merely a visualization component, but an integral functional layer that enables effective human–DT collaboration.

The proposed DT interface is designed to deliver integrated and interpretable representations of the structural system for monitoring and decision support. These include reconstructed full-field states under sparse sensing, uncertainty-aware visualizations, deviations relative to the reference state (State 0/DS0), and prioritized recommendations derived from the decision-support layer. In this way, the interface allows users to rapidly assess structural condition and evolution while preserving access to detailed diagnostic information when required.

A key role of the interface is to explicitly reflect the hybrid nature of the proposed sensing paradigm. Since the framework relies on a limited number of physical sensors complemented by virtual sensor reconstruction, the interface must clearly distinguish between measured and inferred quantities. This distinction is crucial for maintaining epistemic awareness and avoiding overconfidence in reconstructed states. To this end, uncertainty indicators, confidence bands, and reliability cues are embedded in the visualization layer, enabling users to identify well-constrained regions and those that remain weakly observed.

The interface also operationalizes the concept of a reference configuration, termed State 0, which may evolve into a DS0 when re-baselining is justified. Instead of treating observations as isolated snapshots, the interface enables users to evaluate each updated DT state relative to the active reference configuration. Both feature-level variations (e.g., changes in RMS acceleration) and full-field deviations can be visualized, supporting consistent tracking of structural changes over time. As a result, the interface moves beyond static visualization and serves as a platform for temporal interpretation and long-term assessment of structural evolution.

Beyond visualization, the interface plays a central role in decision support. The recommendations presented to users are derived from the outputs of Block 3, which analyze reconstructed structural states, uncertainty estimates, deviations from the reference configuration, and predicted risk indicators to support optimization-based decisions. The interface contextualizes and presents these outputs, which may include suggested sensor locations, inspection priorities, and maintenance actions, in an explainable form. Each recommendation is accompanied by its underlying rationale, such as elevated uncertainty, sensitivity to damage-relevant changes, component criticality, or predicted risk escalation. This transparency is essential for building user trust and enabling informed human intervention.

Another defining feature of the interface is its role in the closed-loop architecture of the proposed framework. User actions, including accepting recommendations, modifying constraints, or initiating inspections, are not external to the system. Instead, they are fed back into the DT as new evidence, influencing subsequent model updates, reconstructions, and optimization cycles. In this way, the interface enables a human-in-the-loop paradigm, where computational intelligence and engineering expertise continuously interact.

The DT interface constitutes a cognitive interaction layer that binds together all functional blocks of the proposed framework. It visualizes the evolving digital model, communicates uncertainty-aware reconstructions, supports baseline-driven longitudinal analysis, and translates optimization outputs into actionable insights. By embedding sparse sensing, explainable recommendations, and closed-loop feedback into a unified environment, the interface elevates the Digital Twin from a computational artifact to an interactive, adaptive management system. This transformation is particularly critical for large-span bridges, where decisions are characterized by high uncertainty, long-term consequences, and limited sensing resources.

## 4. Enabling Technologies and Methods Supporting the Proposed Digital Twin Framework

The technologies discussed in this section are presented as enabling elements that support the implementation of the framework introduced in Section 3.

### 4.1. Technologies Supporting Block 1: Development and Updating of the Digital Model

Block 1 is supported by two closely connected groups of technologies: data-acquisition systems that collect information from the physical bridge, and model-updating tools that use these data to maintain consistency between the physical structure and its digital representation. Recent bridge DT studies identify this coupling between sensing and model updating as a fundamental requirement for practical implementation [37,45,46].

On the data-acquisition side, heterogeneous sensing technologies may be employed depending on bridge type, accessibility, and monitoring objectives. Typical sources include accelerometers, strain gauges, displacement sensors, fiber optic systems, GNSS, LiDAR, and vision-based sensing, which provide complementary information on structural response, deformation, and environmental effects. IoT-enabled monitoring architectures, wireless sensor networks, and cloud-connected platforms further support long-term autonomous operation, remote access, and continuous data transfer, thereby facilitating real-time synchronization with the digital model [47,48].

These observations form the basis for digital model updating, in which discrepancies between simulated and observed structural behavior are reduced by adjusting model parameters, state variables, or response predictions in a physically consistent manner. Depending on the available data, such discrepancies may be evaluated using residuals in measured quantities or derived features, including displacement histories, strain distributions, modal frequencies, and mode shapes. In bridge-oriented DT systems, modal identification, response estimation, and data-driven warning functions have already been incorporated into this synchronization process [49].

Physics-based finite element models remain the core of this updating process because they provide interpretable representations of geometry, material behavior, and boundary conditions. Their consistency with the physical bridge can be improved through deterministic calibration, probabilistic Bayesian updating, or sequential data-assimilation approaches. Methods such as Kalman filtering, particle filtering, and related state-estimation techniques are particularly useful for incorporating streaming measurements and propagating uncertainty over time [46,49].

Beyond direct calibration, historical observations, inspection records, and simulation-generated data can also be used to regularize the updating process and improve robustness under limited or noisy measurements. This is particularly important for existing bridges, where sensing is often sparse and the true structural condition cannot be inferred from real-time data alone. Accordingly, the digital model in Block 1 is treated as a measurement-informed and uncertainty-aware representation that is continuously refined over time. This updated representation provides the basis for defining the initial synchronized reference state, State 0, and for maintaining consistency with the physical bridge during operation [49,50].

### 4.2. Technologies Supporting Block 2: Sensor Placement Optimization, State Reconstruction, and Cyber–Physical Connectivity

Block 2 is enabled by three closely related groups of technologies: sensor placement optimization, state reconstruction for unmeasured structural responses, and digital infrastructures that maintain cyber–physical connectivity. Recent bridge DT studies emphasize that practical deployment depends not only on sensing hardware, but also on how measurements are selected, transmitted, reconstructed, and integrated into the digital environment [37,46,49].

The first group concerns sensor placement optimization. Because dense instrumentation is rarely feasible for existing large-span bridges, only a limited number of sensors can typically be deployed. Their locations must therefore be selected so that the acquired data remain maximally informative for state estimation and damage-sensitive monitoring. In practice, this can be addressed using criteria based on observability, modal participation, Fisher information, entropy, or uncertainty reduction, subject to constraints such as installation cost, accessibility, durability, and maintenance [51,52]. Information-efficient sensing is thus a key requirement for scalable bridge DT implementation [35].

The second group consists of state-reconstruction tools that compensate for incomplete observability. Since only part of the structural response is directly measured, the DT must infer the full-field condition of the bridge from sparse observations. This can be achieved by combining measured data with physics-based constraints, historical observations, and simulation-generated information. Relevant methods include finite-element-based response expansion, modal expansion, reduced-order modeling, Kalman-type state estimation, Bayesian inference, Gaussian process regression, surrogate modeling, autoencoder-based reconstruction, graph-based learning, and physics-informed machine learning [53,54]. Within the proposed framework, these methods support virtual sensing by estimating unmeasured responses together with their associated uncertainty, so that reconstructed states are interpreted as model- and data-supported estimates rather than direct substitutes for physical measurements.

The third group involves the technologies that ensure stable connectivity between the physical and digital domains. These include sensing interfaces, wireless sensor networks, IoT architectures, edge computing, cloud platforms, and interoperable data-management protocols. Together, they support the acquisition, transmission, preprocessing, storage, and synchronization of monitoring data. Edge devices can perform local filtering, feature extraction, and quality control, while cloud-based infrastructures provide scalable storage, distributed computation, and integration with modeling and analytics modules [47].

In the proposed framework, cyber–physical connectivity is defined not merely by data transmission, but by the coordinated integration of sensor placement optimization, state reconstruction, and cyber–physical data infrastructure. Through this combined mechanism, sparse physical measurements are translated into an uncertainty-aware system-level representation, allowing the physical bridge and its Digital Twin to remain functionally coupled even under limited measurement coverage.

### 4.3. Methodological Instantiation of the Virtual Sensing Module

The proposed framework integrates two methodological components that play different roles. The first component is model updating, which establishes the synchronized reference model $M_{0}$ and supports the definition of State 0 in Block 1. In this study, this role is supported by the authors’ previous PSO-based finite element model updating work, where uncertain model parameters were calibrated using experimentally identified modal properties. The second component is virtual sensing, which reconstructs structural responses at un-instrumented locations from sparse physical measurements. Since virtual sensing directly operationalizes the reconstruction operator R(⋅) introduced in Block 2, this subsection focuses on its methodological implementation.

Let S denote the set of available physical sensor locations and U denote the set of un-instrumented or masked virtual sensor locations. For a time window of length T, the sparse physical measurements are arranged as

$$Y_{S}={\left[y_{s}(1),y_{s}(2),\ldots ,y_{s}(T)\right]}^{T}\in R^{T\times m}$$

where $m=\left|S\right|$ is the number of available physical sensor channels. The target output is

$$X_{U}={\left[x_{u}(1),x_{u}(2),\ldots ,x_{u}(T)\right]}^{T}\in R^{T\times q}$$

where $q=\left|U\right|$ is the number of virtual sensor channels to be reconstructed. The reconstruction task is then formulated as

$${\hat{X}}_{U}=R_{CNN-LSTM}(Y_{S},H,M_{0},\theta_{R})$$

where H denotes historical monitoring data, $M_{0}$ is the State 0 model or prior structural information, and $\theta_{R}$ represents the trainable parameters of the reconstruction model.

In the present methodological instantiation, R(⋅) is implemented using a hybrid 1DCNN–LSTM model. The 1DCNN layers extract local temporal and inter-channel features from the sparse sensor signals. For a convolutional filter f with kernel size k, the feature at time index τ is expressed as

$$z_{\tau ,f}=\sum_{i=1}^{k}\sum_{j=1}^{m}W_{i,j,f}^{\left(c\right)}Y_{S}(\tau +i-1,j)+b_{f}^{\left(c\right)}$$

followed by a nonlinear activation,

hτ,f(c)=ReLU(zτ,f)

The extracted feature sequence is then passed to the LSTM layers to capture temporal dependencies in the structural response. Denoting the LSTM hidden state by $h_{\tau }$ the reconstructed virtual sensor response is obtained through a fully connected output layer:

$${\hat{x}}_{U}(\tau )=h_{\tau }W_{fc}+b_{fc}$$

The model is trained using hidden measured channels as ground truth. The loss function is defined as

$$L_{MSE}=\frac{1}{T_{q}}\sum_{\tau =1}^{T}\sum_{j=1}^{q}{\left[x_{U,j}^{true}(\tau )-{\hat{x}}_{U,j}(\tau )\right]}^{2}$$

After training, the reconstructed full-state vector at time t is assembled as

$$\hat{x}(t)=\left[\begin{array}{c} y_{S}(t)\\ {\hat{x}}_{U}(t) \end{array}\right]$$

This formulation directly instantiates the virtual sensing layer of the proposed Digital Twin framework. The physical sensor channels provide direct measurements, while the reconstructed channels provide virtual sensor responses at locations where physical sensors are unavailable. The hidden measured channels are used only for training and validation, not as model inputs during reconstruction.

The implementation procedure is summarized in Algorithm 1:
**Algorithm 1.** The implementation procedure.Input: sparse sensor measurements $Y_{S}$, historical monitoring data H, State 0 model $M_{0}$ masked target channels $X_{U}$ for training and validation.Output: reconstructed virtual sensor responses ${\hat{X}}_{U}$, reconstructed structural state $\hat{x}(t)$ reconstruction reliability indicators.1.  Define the physical sensor set S and the virtual sensor set U2.  Arrange physical sensor data into the time-windowed input matrix Y_S_3.  Mask the channels in U and use them only as ground truth for training and validation.4.  Normalize and preprocess the input data.5.  Pass YS through the 1DCNN layers to extract local temporal and inter-channel features.6.  Pass the extracted features through the LSTM layers to learn temporal dependencies.7.  Use the fully connected layer to reconstruct the virtual sensor responses ${\hat{X}}_{U}$
8.  Train the model by minimizing $L_{MSE}$9.  Validate the reconstructed responses against hidden measured channels using MAE, modal frequency error, and MAC.10.Assemble the reconstructed state $\hat{x}(t)={\left[y_{S}(t),{\hat{x}}_{U}(t)\right]}^{T}$ and pass reconstruction reliability indicators to the optimization and decision-support layer.

Within the proposed framework, this module is not treated as a standalone data-imputation tool. Instead, it instantiates Block 2 by converting sparse physical measurements into an expanded virtual sensing layer. Its outputs provide the reconstructed structural state and reliability indicators required by Block 3 for sensor placement optimization, information-gap detection, inspection prioritization, and maintenance decision support.

### 4.4. Technologies Supporting Block 3: Prediction, Optimization, Decision Support, and Human–DT Interaction

Block 3 is supported by four closely related groups of technologies: predictive methods for forecasting structural evolution, optimization tools for sensing and intervention planning, decision-support methods for prioritizing actions under uncertainty, and interface technologies for communicating these outputs to human users. Recent bridge DT studies increasingly show that practical value lies not only in synchronization and monitoring, but also in transparent support for operation and maintenance decisions [35]. Representative technologies in this block include physics-based prognostic simulation, statistical forecasting, machine learning, reinforcement learning, multi-objective optimization, graph-based decision algorithms, interactive dashboards, WebGIS/WebBIM platforms, and AR/VR-based visualization environments [35,55,56].

The first group comprises predictive technologies, which estimate how the bridge may evolve under future loading, deterioration, and environmental variability. Depending on the application, this function may be supported by physics-based simulations, statistical forecasting models, machine-learning approaches, or hybrid physics-informed learning methods. In bridge-oriented DT systems, such methods can be used for response forecasting, anomaly progression analysis, deterioration trend estimation, and remaining useful life assessment [45].

The second group includes optimization technologies, which convert predicted structural behavior and uncertainty information into monitoring and management actions. In the proposed framework, these tools are relevant to sensor deployment, inspection scheduling, and maintenance planning. Suitable approaches include information-theoretic optimization, multi-objective optimization, Bayesian decision frameworks, and reinforcement learning, particularly when adaptive long-term strategies are required [45].

The third group concerns decision-support methods that synthesize predictions, reconstructed states, and uncertainty into interpretable recommendations. Typical outputs may include suggested sensing configurations, prioritized inspection targets, and recommended intervention strategies. Their rationale may be derived from uncertainty concentration, damage sensitivity, predicted risk escalation, and engineering constraints. In this way, the DT supports comparison among alternatives rather than merely displaying structural information [37].

The fourth group involves human–DT interaction technologies, which ensure that the outputs of prediction and optimization can be understood and used in practice. Advanced visualization, interactive dashboards, web-based asset platforms, and immersive technologies such as augmented reality and virtual reality can improve the accessibility of DT outputs for engineers, inspectors, and infrastructure managers. These tools are particularly important when the system must communicate reconstructed states, uncertainty-aware visualizations, deviations relative to State 0 or DS0, and prioritized recommendations together with their rationale [48].

These technologies allow Block 3 to move beyond condition visualization toward anticipatory, optimization-based, and explainable support for bridge management. The proposed Digital Twin is positioned not as a passive monitoring environment, but as an adaptive decision-support system capable of integrating prediction, optimization, and human interpretation within a unified cyber–physical framework.

## 5. Proof-of-Concept Case Study: Component-Level Evidence for the Proposed Framework

### 5.1. Objective and Scope

The objective of this section is to provide a component-level proof-of-concept for the sparse-sensing Digital Twin framework proposed in this study. In particular, the case study clarifies how two essential operations of the framework can be implemented in practice: first, the establishment of a measurement-informed digital reference model corresponding to State 0; and second, the reconstruction of structural responses at un-instrumented locations using a limited number of physical sensor measurements.

Instead of using a purely numerical benchmark, such as a simply supported beam or an idealized truss model, this proof-of-concept is based on a full-scale laboratory composite plate structure associated with the Thang Long Bridge rehabilitation project. This platform is not a complete in-service bridge. However, it represents a bridge-related structural component with realistic composite action, uncertain boundary conditions, modal behavior, and experimentally measured vibration responses. It therefore offers a more informative proof-of-concept than an idealized numerical benchmark.

The proof-of-concept synthesizes two previously published and experimentally validated studies by the authors. The first study developed a Particle Swarm Optimization (PSO)-based finite element model updating strategy for the composite plate structure [45]. This study supports Block 1 of the proposed framework by demonstrating how a physics-based model can be synchronized with measured dynamic behavior and used as a measurement-informed reference state. The second study developed a hybrid 1DCNN–LSTM model for SHM data reconstruction using the vibration responses collected from the same type of composite plate structure [54]. This study supports Block 2 of the framework by demonstrating how sparse physical measurements can be used to reconstruct responses at un-instrumented or unavailable sensor locations.

It should be emphasized that the present proof-of-concept is not claimed to represent a complete operational Digital Twin deployment. Rather, it provides experimental evidence for the feasibility of the two foundational technical functions required by the proposed framework: measurement-informed model synchronization and sparse-data response reconstruction.

**Figure 7:**
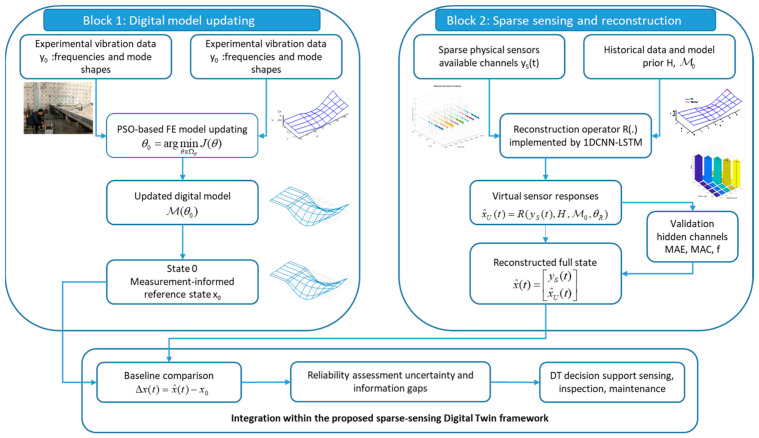
Component-level proof-of-concept workflow of the proposed sparse-sensing Digital Twin framework (based on the work reported in [50,54]).

The PSO-based FE model updating study provides a measurement-informed digital model for establishing State 0, while the 1DCNN–LSTM reconstruction study implements the reconstruction operator R(⋅) to estimate responses at un-instrumented locations from sparse physical measurements (Figure 7). The reconstructed responses are validated using hidden measured channels and modal indicators. Redrawn based on the workflows and datasets reported in [50,54].

### 5.2. Experimental Platform: Full-Scale Composite Plate Structure

The proof-of-concept is based on a full-scale steel–UHPC composite plate structure developed as a laboratory model of a bridge-related cantilever component. The structure consists of an upper composite deck, an I-section transverse beam, and a steel box girder supported by steel blocks. One end of the plate is fixed to the laboratory wall to reproduce a cantilever-type boundary condition. The composite deck consists of a 14 mm steel plate and a 65 mm UHPC layer connected through welded studs. Transverse stiffeners, an I-section beam, and a box girder are incorporated to reproduce the mechanical complexity of the bridge-related component.

The nominal dimensions of the structure are approximately 3.3 × 7.25 × 0.865 m. The measurement grid contains 35 points arranged on the plate surface. Due to the limited number of available sensors and data-acquisition channels, only eight accelerometers are used in each measurement round. To obtain spatially distributed vibration information, the experimental campaign adopts a reference–roving sensor strategy. Three points, namely 03, 06, and 35, are used as reference locations, while the remaining sensors are moved across the measurement grid in different sub-measurements.

This experimental configuration is well aligned with the sparse-sensing assumption of the proposed Digital Twin framework. The complete structural response cannot be measured simultaneously at all locations. Instead, only a limited number of physical sensor channels are available in each measurement round. This makes the composite plate dataset suitable for demonstrating how sparse measurements can be used to establish a reference digital model and reconstruct unmeasured responses through virtual sensing.

### 5.3. Measurement-Informed Model Updating for State 0

The first component of the proof-of-concept concerns the establishment of a synchronized digital reference model. In the authors’ previous work [50], an initial finite element model of the composite plate structure was developed using available geometric, material, and boundary-condition information. However, as is typical for existing bridge components and composite structures, several parameters remained uncertain, including material stiffness, mass density, and support stiffness.

To reduce the discrepancy between numerical predictions and experimental observations, the uncertain parameters were updated using PSO. The updating process minimized an objective function combining modal frequency discrepancies and mode-shape correlation through the Modal Assurance Criterion (MAC). In general form, the model updating problem can be expressed as

$$\theta_{0}=\arg\underset{\theta \in \Omega_{\theta }}{\min}\;J(\theta )$$

where θ denotes the vector of uncertain model parameters, Ω_θ_ is the admissible parameter domain, and J(θ) represents the mismatch between numerical and experimental modal properties.

The PSO-based updating procedure substantially improved the agreement between the FE model and the measured structural behavior. The initial FE model exhibited frequency errors ranging from approximately 4.92% to 7.67%. After updating, these errors were reduced to 0.12–1.39%, while the MAC values increased to 0.91–0.96 for the first five modes [50]. The updated model therefore provides a measurement-informed digital representation of the composite plate at the time of testing.

From the perspective of the proposed Digital Twin framework, this updated model provides a practical basis for defining State 0. The synchronized reference state can be written as

$$x_{0}=S(M(\theta_{0}),y_{0})$$

where M(θ_0_) denotes the updated digital model, y0 denotes the experimental measurements used for synchronization, and S(⋅)represents the synchronization process. In this interpretation, State 0 is not an idealized pristine condition. Rather, it is a validated reference configuration established from measured structural behavior and an updated physics-based model.

This first component directly supports Block 1 of the proposed framework. It demonstrates that a physics-based model can be transformed from an initial engineering approximation into a synchronized digital representation suitable for baseline definition and subsequent structural-state comparison.

### 5.4. Sparse Sensor Definition and Virtual Sensor Reconstruction

**Figure 8:**
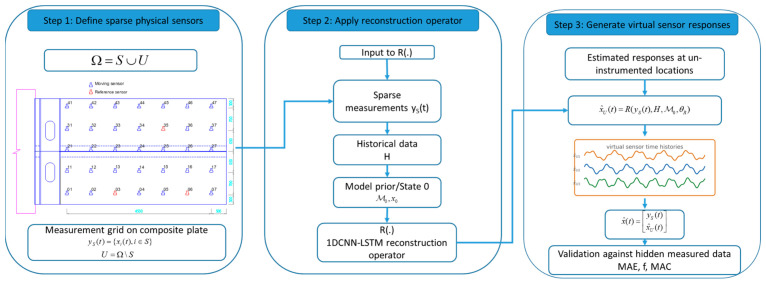
Sparse sensing emulation and virtual sensor reconstruction (based on the work reported in [54]).

The second component of the proof-of-concept addresses sparse sensing and virtual sensor reconstruction. In the authors’ previous SHM data reconstruction study [54], acceleration responses from the composite plate structure were used to train and evaluate a hybrid 1DCNN–LSTM model (Figure 8). The original dataset contained multiple measured sensor channels, but sparse sensing was emulated by retaining only a subset of these channels as available physical measurements and masking the remaining channels. The masked channels were then treated as un-instrumented or virtual sensor locations.

Let

Ω=S∪U;S∪U=Ø

denote the complete set of response locations considered in the dataset, where S is the subset of physical sensor locations and U is the subset of un-instrumented locations. The available physical measurements are denoted as

$$y_{S}(t)=\{x_{i}(t),i\in S\}$$

The objective of the reconstruction process is to estimate the responses at the un-instrumented locations U:

$$\begin{array}{l} {\hat{x}}_{U}(t)=R(y_{S}(t),H,M_{0},\theta_{R})\\ M_{0},x_{0} \end{array}$$

where R(⋅) is the reconstruction operator, H denotes historical monitoring data, M0 represents prior model-based information or the synchronized reference model, and θ_R_ denotes the trained parameters of the reconstruction model. In this proof-of-concept, R(⋅) is implemented using the hybrid 1DCNN–LSTM model developed in [54].

The reconstructed full-state vector is then assembled as

$$\hat{x}(t)=\left[\begin{array}{c} y_{S}(t)\\ {\hat{x}}_{U}(t) \end{array}\right]$$

This formulation directly operationalizes the sparse-sensing concept of the proposed Digital Twin framework. A limited number of measured physical sensor channels are used to infer responses at locations where no physical sensors are assumed to be available. The hidden measured channels are used only for validation and are not provided to the reconstruction model during inference.

A subset S of measured locations is retained as physical sensor input, while the remaining locations U = Ω∖S are masked and treated as un-instrumented virtual sensor locations. The reconstruction operator R(⋅), implemented using the 1DCNN–LSTM model, estimates ${\hat{x}}_{U}(t)$ from y_S_(t), historical data H, and the model prior M0. The hidden measured responses at U are used only for proof-of-concept validation through MAE, modal frequency error, and MAC. Redrawn based on the measurement grid and reconstruction scenarios reported in [50,54].

### 5.5. Implementation of the Reconstruction Operator R(⋅)

The reconstruction operator R(⋅) is implemented using a hybrid architecture that combines one-dimensional Convolutional Neural Networks and Long Short-Term Memory networks. The 1DCNN layers are used to extract local and spatially correlated features from the available sensor signals, while the LSTM layers capture temporal dependencies in the dynamic response. This combination is suitable for SHM data because structural vibration responses contain both spatial correlations among sensor locations and temporal dependencies associated with dynamic excitation.

In the single-channel data-loss scenario, seven sensor channels are retained as available physical measurements, while one channel is masked and reconstructed. In multi-channel data-loss scenarios, the number of unavailable channels is progressively increased from two to seven. Correspondingly, the number of input channels decreases while the number of reconstructed output channels increases. This procedure provides a direct experimental analogy to sparse sensing: the fewer the available physical sensors, the more the Digital Twin must rely on virtual reconstruction.

The reconstruction quality is evaluated by comparing the reconstructed responses with the hidden measured responses. Three complementary indicators are used. The first is the Mean Absolute Error:

$$MAE=\frac{1}{T\left|U\right|}\sum_{t=1}^{T}\sum_{i\in U}\left|x_{i}(t)-{\hat{x}}_{i}(t)\right|$$

The second is the modal frequency error:

$$\varepsilon_{f,k}=\frac{\left|f_{k}^{real}-f_{k}^{rec}\right|}{f_{k}^{real}}\times 100\%$$

where $f_{k}^{real}$ and $f_{k}^{rec}$ denote the k-th modal frequency obtained from the real and reconstructed datasets, respectively. The third is the Modal Assurance Criterion:

$$MAC(\phi_{k}^{real},\phi_{k}^{rec})=\frac{{\left|{\left(\phi_{k}^{real}\right)}^{T}\phi_{k}^{rec}\right|}^{2}}{\left[\phi_{k}^{real}{)}^{T}\phi_{k}^{real}\right]\left[\phi_{k}^{rec}{)}^{T}\phi_{k}^{rec}\right]}$$

These indicators assess reconstruction quality at both signal and structural-dynamics levels. This is important because a reconstructed signal may appear accurate in the time domain but still distort modal characteristics relevant to SHM. By using both time-domain and modal indicators, the proof-of-concept evaluates whether virtual sensor outputs preserve the dynamic behavior of the physical structure.

### 5.6. Reconstruction Results for the Composite Plate Structure

The single-channel reconstruction results demonstrate that the hybrid 1DCNN–LSTM operator can accurately recover a hidden sensor response from the remaining measured channels. For the composite plate dataset, the modal frequency errors between the reconstructed and real datasets ranged from approximately 1.024% to 1.162%, while the MAC values ranged from 0.986 to 0.997 for the first five modes [54]. These results indicate that the reconstructed responses are not only close to the measured signals in the time domain but also consistent with the structural dynamic characteristics of the original dataset.

The multi-channel reconstruction results provide additional insight into the limits of sparse sensing. When two to four sensor channels are unavailable, the reconstructed responses retain acceptable modal consistency, with MAC values remaining above 0.9 [54]. However, the reconstruction reliability decreases as the number of unavailable channels increases. In particular, when five out of eight sensor channels are reconstructed, the highest MAC value decreases to 0.789. When six and seven channels are unavailable, the corresponding MAC values further decrease to 0.692 and 0.519, respectively [54].

Table 3 summarizes the main performance indicators obtained from the composite plate reconstruction workflow. The sparse-measurement condition is emulated by retaining only a subset of measured channels as physical sensor inputs and treating the remaining channels as virtual sensor targets. Reconstruction quality is then evaluated by comparing the reconstructed responses with the hidden measured channels using modal frequency error and MAC. These indicators assess not only signal-level reconstruction but also whether the reconstructed data preserve the dynamic characteristics required for SHM.

**Figure 9:**
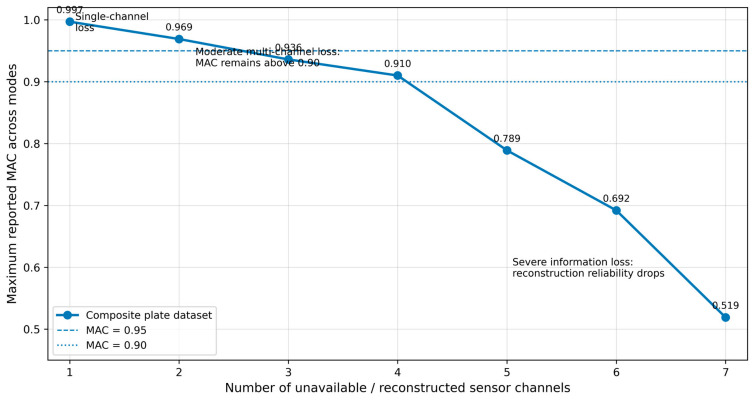
Reconstruction reliability under increasing information loss for the composite plate structure (based on the work reported in [54]).

These results support three observations (Figure 9). First, the proposed reconstruction operator R(⋅) can be practically instantiated using a trained data-driven model. Second, a limited number of physical sensor measurements can be used to reconstruct responses at un-instrumented locations with high modal consistency, provided that the remaining measurements contain sufficient structural information. Third, reconstruction reliability decreases when the available physical measurements become too sparse. This confirms that virtual sensing cannot fully compensate for arbitrary information loss and must be accompanied by uncertainty quantification and sensor placement optimization.

Based on the established State 0 model and the demonstrated reconstruction capability, an illustrative stiffness-reduction scenario is introduced below to show how reconstructed state deviations can be used for damage indication under sparse sensing conditions.

### 5.7. Illustrative Damage Scenario Based on Stiffness Reduction

To illustrate how the proposed framework can support damage indication, a simple numerical stiffness-reduction scenario is considered using the composite plate structure. The purpose of this example is not to provide experimental damage validation, but to show how a local structural change can be reflected through deviations from State 0.

The updated finite element model obtained from the model-updating process is used as the State 0 reference model. A local damage scenario is simulated by reducing the stiffness of a selected region Ω_d_ of the plate:

$$K^{d}=K^{0}-\eta K_{\Omega d}$$

where $K^{0}$ is the stiffness matrix of the State 0 model, $K_{\Omega d}$ is the stiffness contribution of the selected damaged region, and η is the stiffness reduction ratio.

The damaged model is then used to generate structural responses. Only a limited number of responses are treated as sparse physical sensor measurements, while the remaining responses are reconstructed using the operator R(⋅). The reconstructed damaged state is denoted as ${\hat{x}}^{d}(t)$. Damage indication is performed by comparing this reconstructed state with the State 0 response:

$$\Delta x(t)={\hat{x}}^{d}(t)-x_{0}(t)$$

A simple damage-sensitive deviation index can be defined for each location j as

$$D_{j}=\frac{RMS(x_{j}(t))}{\sigma_{0,j}+\sqrt{\sum_{x,jj}^{d}+\varepsilon }}$$

where $\sigma_{0,j}$ is the baseline variability at location j, $\sum_{x,jj}^{d}$ is the reconstruction uncertainty, and ε is a small regularization term. Locations with larger $D_{j}$ values indicate stronger deviations from the State 0 condition and are therefore flagged as potential damage-sensitive regions.

This example shows how the proposed framework can use the reconstructed state deviation Δx(t) to support damage localization under sparse sensing conditions. The local stiffness reduction produces a deviation pattern that becomes concentrated around the damaged region. The resulting deviation map can then be used to guide inspection or further model reassessment.

**Figure 10:**
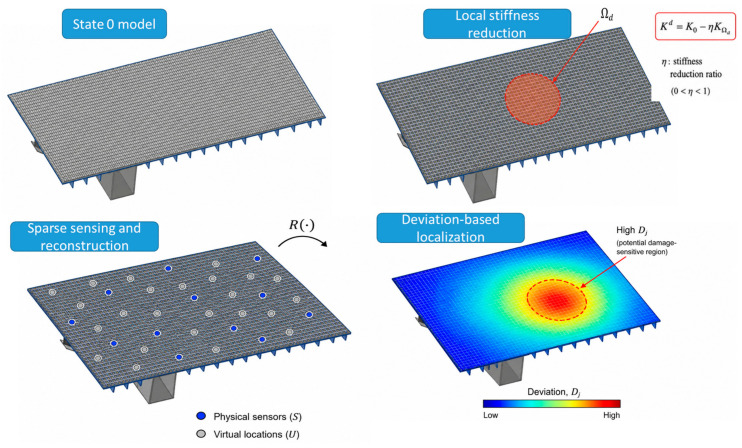
Illustrative stiffness-reduction scenario for deviation-based damage localization.

A local stiffness reduction is introduced into a selected region of the State 0 composite plate model. Sparse responses from the damaged state are used as physical measurements, while the remaining responses are reconstructed through R(⋅). The deviation field $\Delta x(t)={\hat{x}}^{d}(t)-x_{0}(t)$ is converted into a damage-sensitive index Dj, allowing regions with abnormal deviations to be flagged for inspection. Figure 10 is an illustrative numerical scenario rather than an experimental damage validation.

The same deviation and uncertainty maps can also support a one-step sensor placement recommendation. Let C denote the set of feasible candidate locations, excluding already instrumented or inaccessible points. For each candidate location i, the DT estimates the expected reduction in reconstruction uncertainty if an additional sensor is installed at that location:

$$\Delta J=tr(W\sum_{x})-tr(\sum_{x}^{+i})-\lambda_{c}c_{i}$$

where $\sum_{x}$ is the current time-averaged reconstruction uncertainty, $\sum_{x}^{+i}$ is the expected uncertainty after adding a sensor at location i, W emphasizes critical or damage-sensitive regions, ci is the installation cost, and λc is a cost-penalty coefficient. The recommended sensor location is then selected as

$$i*=\arg\underset{i\in C}{\max}\;\Delta J_{i}$$

Thus, one optimization iteration consists of identifying high-uncertainty or high-deviation regions, evaluating the marginal uncertainty reduction associated with feasible candidate locations, and recommending the location that provides the largest information benefit under practical constraints. After updating the sensing configuration, the reconstruction uncertainty can be recalculated and the process repeated.

### 5.8. Integrated Interpretation Within the Proposed Digital Twin Framework

The two components of the proof-of-concept correspond directly to the first two functional blocks of the proposed framework. The PSO-based FE model updating study supports Block 1 by demonstrating that a physics-based digital model can be synchronized with measured dynamic behavior and used to establish State 0. The 1DCNN–LSTM reconstruction study supports Block 2 by demonstrating that sparse physical measurements can be expanded into virtual sensor responses through the reconstruction operator R(⋅).

The integrated proof-of-concept workflow can be summarized as follows. First, experimental vibration data are used to update the finite element model, producing a synchronized digital representation $M_{\theta_{0}}$. Second, this updated model provides the basis for defining the reference state x_0_. Third, a sparse set of physical sensor locations S is retained from the measurement grid. Fourth, the remaining locations U are treated as un-instrumented virtual sensor locations. Fifth, the reconstruction operator R(⋅) estimates ${\hat{x}}_{U}(t)$ from the available measurements y_S_(t). Finally, the reconstructed state

$$\hat{x}(t)=\left[\begin{array}{c} y_{S}(t)\\ {\hat{x}}_{U}(t) \end{array}\right]$$

can be interpreted relative to the reference state:

$$\Delta x(t)=\hat{x}(t)-x_{0}$$

This formulation links sparse sensing, virtual reconstruction, and baseline-aware interpretation into a unified Digital Twin logic. The reconstructed state provides an expanded representation of the structural response, while the reference state provides the temporal anchor required for longitudinal monitoring.

The degradation of reconstruction reliability under severe sensor loss is also relevant to Block 3 of the proposed framework. When reconstruction accuracy deteriorates due to insufficient input information, the Digital Twin can interpret this condition as an information gap. This information gap can then motivate sensor placement optimization, sensor redundancy planning, inspection prioritization, or temporary adjustment of the monitoring strategy. In this sense, the proof-of-concept does not only demonstrate reconstruction capability; it also provides empirical motivation for the optimization and decision-support layer of the proposed framework.

### 5.9. Limitations of the Proof-of-Concept

Although the proof-of-concept provides experimental support for the proposed framework, it remains a component-level demonstration rather than a full operational Digital Twin implementation. The composite plate structure is a bridge-related experimental component, not a complete in-service large-span bridge. Moreover, the model updating and data reconstruction components were validated as separate technical modules rather than as a continuously integrated cyber–physical loop.

Several aspects therefore require further development before full-scale deployment. First, the model updating and reconstruction modules should be integrated into a continuous online workflow. Second, reconstructed responses should be accompanied by explicit uncertainty estimates rather than being interpreted as deterministic substitutes for direct measurements. Third, environmental and operational variability should be incorporated to avoid confusing normal response variability with structural change. Fourth, the optimization layer should be implemented to determine the most informative sensor locations and inspection priorities under practical constraints. Finally, the human–DT interface should be tested in realistic bridge management scenarios.

Despite these limitations, the proof-of-concept demonstrates that the core technical logic of the proposed framework is feasible. A measurement-informed digital model can be established through PSO-based FE model updating, and sparse physical measurements can be used to reconstruct responses at un-instrumented locations through the 1DCNN–LSTM reconstruction operator. These two capabilities provide the experimental basis for the proposed sparse-sensing Digital Twin framework.

## 6. Challenges and Future Directions

### 6.1. Positioning Relative to Recent Bridge Digital Twin Frameworks

Table 4 compares it with several recent bridge Digital Twin frameworks. The comparison focuses on aspects that are particularly relevant to sparse-sensing SHM applications, including sensing-density assumptions, reference baseline management, virtual sensing or full-field reconstruction, uncertainty representation, optimization and decision-support functions, and the type of case-study validation.

Several recent bridge DT studies have made important progress in integrating SHM data, IoT platforms, bridge information models, and full-field monitoring concepts. Compared with these studies, the proposed framework differs in four main aspects. First, sparse sensing is treated as a design condition rather than a secondary limitation. Second, the framework introduces an explicit reference-state mechanism through State 0 and DS0, enabling longitudinal interpretation of reconstructed states. Third, the reconstruction operator R(⋅) is linked to uncertainty-aware virtual sensing, enabling measured and reconstructed quantities to be interpreted with different levels of confidence. Fourth, the optimization and decision-support layer is formulated explicitly through sensor placement, priority ranking, and risk-trajectory metrics. These differences do not imply that the proposed framework is a fully deployed operational DT. Rather, they define its intended contribution as a sparse-sensing-aware, decision-oriented DT architecture, supported by a component-level proof of concept.

### 6.2. Challenges

Despite the conceptual coherence of the proposed framework, several challenges must be addressed before it can be implemented reliably for existing large-span bridges. These challenges arise from the specific requirements of the proposed framework. They include the construction of a physically meaningful digital model, cyber–physical synchronization under sparse sensing, and the translation of reconstructed states into trustworthy decision support.

A first challenge concerns Block 1, which requires the development and continuous updating of a digital model that remains both physically interpretable and operationally representative over time. For existing bridges, the information needed to construct such a model is often incomplete or uncertain, particularly regarding material properties, boundary conditions, prior interventions, and accumulated deterioration. As a result, maintaining consistency between simulated and observed structural behavior is inherently difficult. This challenge becomes even more critical when the synchronized reference state, State 0, is used as the basis for subsequent comparison. If the initial synchronization is weak or if the reference configuration is poorly defined, the reliability of all later assessments may be compromised. Moreover, once major interventions or significant condition changes occur, the framework must determine when and how to rebaseline into a Dynamic State 0 (DS0) without losing temporal continuity.

A second challenge arises in Block 2, where the framework explicitly assumes sparse sensing rather than dense instrumentation. Although this assumption improves practical feasibility, it also introduces a fundamental observability problem: only a limited portion of the structural response is directly measured, while the remaining information must be inferred. Consequently, the reliability of full-field state reconstruction depends strongly on sensor placement, reconstruction methodology, and uncertainty quantification. Errors in any of these components may propagate through the Digital Twin, distorting the inferred structural condition. In addition, maintaining a stable cyber–physical connection is not merely about transmitting measurements from the bridge to the digital platform. It also requires robust data quality control, resilience to sensor malfunction and communication loss, and a clear distinction between directly measured quantities and computationally reconstructed states. Without such safeguards, the cyber–physical loop may appear conceptually complete while remaining operationally fragile.

A third challenge arises in Block 3, where reconstructed states and uncertainty estimates are expected to support prediction, optimization, and decision-making. In principle, this is one of the main distinguishing features of the proposed framework; in practice, however, it is also one of the most demanding. Prediction models must remain sufficiently accurate under changing structural and environmental conditions, while optimization routines must operate under realistic engineering constraints such as cost, accessibility, traffic disruption, and maintenance feasibility. At the same time, recommendations related to sensor deployment, inspection scheduling, or maintenance planning must remain explainable and transparent to human users. If the rationale behind these recommendations is not clear, the system may fail to gain the trust required for adoption in bridge management practice.

A final challenge lies in the integration of all three blocks into a unified operational system. The proposed framework depends on the coordinated interaction of sensing technologies, digital modeling tools, reconstruction methods, uncertainty-aware analytics, optimization procedures, and human-centered interfaces. Each component may be feasible in isolation, yet their combined implementation introduces substantial methodological, computational, and organizational complexity. Therefore, the main challenge is not only to improve individual technologies but also to ensure that they work together consistently within a scalable, robust, and interpretable Digital Twin architecture for existing large-span bridges.

### 6.3. Future Directions

Future research should focus on strengthening the practical realization of the proposed framework rather than treating its components as isolated technological advances. In particular, progress is needed in methods that improve the physical reliability of the digital model, the robustness of sparse-sensing-based state reconstruction, and the transparency of prediction and decision support under uncertainty.

For Block 1, an important research direction is the development of more reliable strategies for digital model initialization, updating, and reference-state management in existing bridges. This includes improved methods for incorporating incomplete design information, inspection records, and long-term monitoring data into model calibration. Particular attention should also be given to uncertainty-aware synchronization and to the formal definition of re-baselining criteria for transitioning from State 0 to Dynamic State 0 (DS0). Future work should clarify under what structural, operational, or intervention-related conditions a new reference configuration can be established without compromising the consistency of temporal assessment.

For Block 2, future studies should advance sparse-sensing methodologies that remain effective under real-world constraints. This includes more rigorous approaches to sensor placement optimization, as well as more robust reconstruction methods capable of inferring full-field structural states from incomplete and noisy observations. Research is also needed on uncertainty-aware virtual sensing, so that reconstructed states are accompanied by reliable confidence information rather than being treated as deterministic estimates. In parallel, cyber–physical connectivity should be made more resilient through improved data quality control, fault-tolerant communication architectures, and adaptive mechanisms for handling missing or degraded measurements. These developments are essential if sparse sensing is to become a practical foundation for bridge Digital Twins rather than merely a conceptual simplification.

For Block 3, future directions should concentrate on making prediction and decision support both more reliable and more actionable. This requires predictive models that remain robust under changing structural conditions and environmental variability, as well as optimization methods that explicitly account for engineering constraints such as cost, accessibility, intervention timing, and service disruption. Equally important is the need for explainable recommendation mechanisms, so that outputs related to sensing, inspection, and maintenance can be interpreted and trusted by engineers and infrastructure managers. Future work on human–DT interaction should therefore move beyond visualization alone and focus on interfaces that communicate not only structural states, but also uncertainty, risk, decision rationale, and the implications of alternative management actions.

At the system level, a major future direction is the integrated validation of the framework through realistic bridge case studies. Such validation should not be limited to demonstrating individual functions, such as model updating or response reconstruction, in isolation. Instead, it should assess whether the full chain of the proposed framework—from sensing and synchronization to reconstruction, prediction, optimization, and human-centered interpretation—can operate consistently and deliver measurable value for bridge management. In this sense, future research should aim to transform the framework from a conceptually sound architecture into a deployable, scalable, and decision-relevant Digital Twin system for existing large-span bridges.

## 7. Conclusions

This paper proposed a sparse-sensing-aware Digital Twin framework for structural health monitoring and lifecycle management of existing large-span bridges. The framework integrates three main functions: digital model updating, virtual sensing under sparse measurements, and optimization-oriented decision support. It also introduces State 0 and Dynamic State 0 (DS0) as reference configurations for longitudinal state comparison.

A component-level proof-of-concept was presented using a bridge-related composite plate structure. The case study illustrated how a measurement-informed digital model can support State 0 definition and how sparse measurements can be expanded into virtual sensor responses using a 1DCNN–LSTM reconstruction operator. For the composite plate dataset, single-channel reconstruction produced modal frequency errors of approximately 1.024–1.162% and MAC values of 0.986–0.997. Under moderate multi-channel loss, where two to four channels were reconstructed, MAC values remained above 0.90. Under severe information loss, reliability decreased, with maximum MAC values of 0.789, 0.692, and 0.519 when five, six, and seven channels were unavailable, respectively.

These results provide preliminary evidence for the feasibility of the virtual sensing component. They also show that sparse reconstruction has practical limits and must be supported by uncertainty quantification and sensor placement optimization. An illustrative stiffness-reduction scenario further demonstrated how reconstructed state deviations can be converted into a damage-sensitive indicator. This example clarifies the operational logic of the framework but does not constitute experimental damage validation.

The proposed framework should therefore be interpreted as a structured DT architecture supported by component-level evidence, rather than as a fully validated operational bridge DT. Future work should focus on full-system validation using long-term bridge monitoring data. Key priorities include continuous cyber–physical synchronization, uncertainty-aware reconstruction and prediction, State 0/DS0 re-baselining criteria, optimization-based sensor placement, and human-in-the-loop decision support.

## Figures and Tables

**Table 1:** Comparison among BIM, SHM, smart infrastructure systems, and Digital Twin (DT) for bridge lifecycle management.

Aspect	BIM (Building Information Modeling)	SHM (Structural Health Monitoring)	Smart Infrastructure Systems	Digital Twin (DT)
Primary purpose	Design, documentation, and asset information management	Condition monitoring and damage detection	Integrated sensing, communication, and automated infrastructure operation	Continuous synchronization between physical asset and digital representation for prediction and decision support
Temporal nature	Largely static or discretely updated	Continuous or periodic data acquisition	Continuous operation with real-time or near-real-time responses	Continuous, time-evolving
Data source	Design models, construction records, inspection reports	Sensor measurements (strain, acceleration, displacement,…)	Sensor networks, communication systems, control platforms, and operational data	Multi-source: sensor data, physics-based models, historical records, inspections
Level of physical fidelity	High geometric/detail fidelity	Limited to instrumented locations	System-aware but often function-oriented rather than full structural representation	Full-field, system-level representation (measured + reconstructed states)
Observability	Complete in geometry, limited in physics	Sparse, sensor-dependent	Moderate, depending on sensing and automation infrastructure	Enhanced via virtual sensing and data–model fusion
Uncertainty handling	Usually deterministic	Often implicit or heuristic	Partially addressed, often application-specific	Explicit, probabilistic, and quantified
Predictive capability	Very limited	Short-term trend analysis	Limited to operational forecasting or rule-based anticipation	Long-term prognosis and scenario simulation
Prescriptive capability	None	Limited (alarm-based)	Rule-based or semi-automated operational response	Decision-oriented (maintenance, retrofit, inspection planning)
Feedback loop	No closed-loop	Weak or manual	Operational feedback loop, typically localized	Closed-loop with continuous updating
Typical output	3D model, drawings, metadata	Damage indicators, alerts, trends	System status, control actions, operational alerts	State estimates, confidence intervals, risk-informed decisions
Lifecycle coverage	Design–construction–handover	Operation and maintenance	Primarily operation and service management	Full lifecycle
Adaptability	Low	Medium	Medium to high	High

**Table 2:** Functional progression across four Digital Twin implementation stages.

Maturity Level	Description	Core Functional Capability	Typical Applications	Main Advantages	Main Limitations
Level 1: Digital Model	A static or quasi-static digital representation of the physical asset with no continuous synchronization	Geometric and physics-based representation	Design-stage simulation, documentation, offline structural analysis	High interpretability, strong physical basis, useful for design and assessment	No real-time updating, limited operational relevance, no closed-loop capability
Level 2: Synchronized Digital Twin	A digital representation updated periodically or continuously using monitoring data	State synchronization, model updating, anomaly detection	Structural health monitoring, condition assessment, dashboard-based monitoring	Reflects current structural condition, supports near-real-time awareness	Often descriptive rather than predictive, limited adaptation, usually dependent on measured locations
Level 3: Predictive Digital Twin	A synchronized DT extended with forecasting and scenario-analysis capabilities	Prognosis, trend prediction, remaining useful life estimation, what-if simulation	Degradation forecasting, risk projection, maintenance planning support	Enables proactive management and future-oriented assessment	Predictions may remain open-loop, uncertainty may not be fully propagated, monitoring strategy is usually fixed
Level 4: Prescriptive and Adaptive Digital Twin	A DT that not only predicts system evolution but also evaluates information gaps and recommends or triggers actions through feedback	Closed-loop optimization, uncertainty-aware inference, adaptive sensing, decision support	Sensor placement optimization, inspection scheduling, maintenance prioritization, lifecycle management	Supports actionable decision-making, improves information efficiency, adapts to evolving conditions	High implementation complexity, stronger computational requirements, greater need for interoperability and trustworthy human–DT interaction

**Table 3:** Quantitative summary of the sparse-measurement reconstruction workflow for the composite plate structure.

Workflow Stage	Scenario	Available Physical Sensor Channels	Reconstructed Virtual Sensor Channels	Quantitative Indicator	Result	Interpretation
Sparse measurement → virtual reconstruction	Single-channel loss	7	1	Modal frequency error	1.024–1.162%	Reconstructed response preserves modal frequencies with low error
Sparse measurement → virtual reconstruction	Single-channel loss	7	1	MAC	0.986–0.997	Very high modal consistency between real and reconstructed data
Sparse measurement → virtual reconstruction	Moderate multi-channel loss	6–4	2–4	MAC	>0.90	Reconstruction remains reliable under moderate information loss
Sparse measurement → virtual reconstruction	Severe multi-channel loss	3	5	Maximum MAC	0.789	Reliability decreases when available information becomes insufficient
Sparse measurement → virtual reconstruction	Severe multi-channel loss	2	6	Maximum MAC	0.692	Strong degradation in reconstruction reliability
Sparse measurement → virtual reconstruction	Severe multi-channel loss	1	7	Maximum MAC	0.519	Sparse input becomes insufficient for reliable reconstruction
State comparison/damage indication	Illustrative stiffness reduction	Sparse set (S)	Virtual set (U)	Deviation index (Dj)	High (Dj) near (Ωd)	Localized deviation from State 0 indicates potential damage-sensitive region

**Table 4:** Targeted comparison between recent bridge Digital Twin frameworks and the proposed framework.

Study/Framework	Primary Focus	Sensing-Density Assumption	Reference Baseline Management	Virtual Sensing/Full-Field Reconstruction	Uncertainty Handling	Optimization/Decision-Support Function	Case-Study Validation
Costin et al. [57]	Integration of existing technologies for bridge SHM, management, operation, and maintenance	Assumes availability of SHM and asset-management data; sparse sensing is not treated as a central design constraint	No explicit State 0/DS0-type reference baseline mechanism	Mainly framework-level integration; full-field virtual sensing is not the central focus	Discussed generally through SHM data quality and lifecycle management	Supports management and maintenance decisions, but optimization formulation is not explicitly developed	Framework-oriented; focused on technology integration and bridge management context
Armijo and Zamora-Sánchez [58]	IoT-based SHM integration with a railway bridge DT	Uses an instrumented railway bridge and IoT monitoring architecture	No explicit dynamic baseline management comparable to State 0/DS0	Focuses on sensor-data integration and DT connectivity rather than sparse-to-full-field reconstruction	Mainly related to monitoring data and system operation	Supports monitoring and visualization; limited explicit optimization or prescriptive decision logic	Demonstrated through a railway bridge case study
Sun et al. [59]	Integration of FEM, bridge information model, IoT, and hybrid monitoring for full-field sensing	Uses spatially discrete data and expands them through model-data integration	Reference-state management is not the main contribution	Strong emphasis on full-field virtual sensing and data expansion	Partly addressed through model-data fusion, but uncertainty-driven decision rules are limited	Focuses on full-field monitoring; optimization/decision support is less explicitly formulated	Validated through bridge-oriented full-field monitoring demonstrations
Proposed framework	Sparse-sensing-aware DT for existing large-span bridge SHM	Sparse sensing is an explicit design assumption; limited physical sensors are complemented by virtual sensors	Explicit State 0 and Dynamic State 0 reference management for longitudinal comparison	(R(.)-based virtual sensing; proof-of-concept instantiated using 1DCNN–LSTM reconstruction	Explicit uncertainty term $\sum_{x}(t)$; reliability interpreted through reconstruction uncertainty and MAC degradation	Includes mathematical formulations for sensor placement, priority ranking, risk trajectories, and one-step sensor recommendation	Component-level proof-of-concept using composite plate model updating and reconstruction; full bridge deployment remains future work

## Data Availability

The source code for the Digital Twin interface is available from the corresponding author upon reasonable request.
